# Utility of T-Cell Interferon-γ Release Assays for Diagnosing Tuberculous Serositis: A Prospective Study in Beijing, China

**DOI:** 10.1371/journal.pone.0085030

**Published:** 2014-01-09

**Authors:** Lifan Zhang, Yueqiu Zhang, Xiaochun Shi, Yao Zhang, Guohua Deng, Ajit Lalvani, Xiaoqing Liu

**Affiliations:** 1 Department of Infectious Diseases, Peking Union Medical College Hospital, Chinese Academy of Medical Sciences & Peking Union Medical College, Beijing, China; 2 Clinical Epidemiology Unit, Peking Union Medical College, International Clinical Epidemiology Network, Beijing, China; 3 Department of Respiratory Medicine, National Heart and Lung Institute, Imperial College London, London, United Kingdom; Institut de Pharmacologie et de Biologie Structurale, France

## Abstract

**Background:**

Diagnosis of tuberculous serositis remains a challenge. The aim of this study was to evaluate the diagnostic efficiency of T-SPOT.TB on serous effusion mononuclear cells (SEMC) for diagnosing tuberculous serositis in a high TB burden area.

**Methods:**

The present prospective study enrolled patients with suspected tuberculous serositis in a tertiary referral hospital in Beijing, China, to investigate the diagnostic sensitivity, specificity, predictive value (PV), and likelihood ratio(LR) of these tests. Clinical assessment, T-SPOT.TB on SEMC, and T-SPOT.TB on PBMC were performed. Test results were compared with the final confirmed diagnosis.

**Results:**

Of the 187 participants, 74 (39.6%) were microbiologically or clinically diagnosed as tuberculous serositis and 93(49.7%) were ruled out. The remaining 20 (10.7%) patients were clinically indeterminate and excluded from the final analysis. Compared to that on PBMC, T-SPOT.TB on SEMC showed higher sensitivity (91.9%vs73.0%, P = 0.002), specificity (87.1%vs.73.1%, P = 0.017), PPV (85.0%vs.68.4%, P = 0.013), NPV (93.1%vs.77.3%, P = 0.003), LR+ (7.12vs.2.72) and LR- (0.09vs.0.37), respectively. The frequencies of spot forming cells (SFCs) for T-SPOT.TB on SEMC were 636 per million SEMC (IQR, 143–3443) in patients with tuberculous serositis, which were 4.6-fold (IQR, 1.3–14.3) higher than those of PBMC. By ROC curve analysis, a cut-off value of 56 SFCs per million SEMC for T-SPOT.TB on SEMC showed a sensitivity of 90.5% and specificity of 89.2% for the diagnosis of tuberculous serositis.

**Conclusions:**

T-SPOT.TB on SEMC could be an accurate diagnostic method for tuberculous serositis in TB endemic settings. And 56 SFCs per million SEMC might be the optimal cut-off value to diagnose tuberculous serositis.

## Introduction

China ranks second among the 22 high burden countries for tuberculosis (TB). According to the fifth national TB surveillance in 2010, 1.3 million new cases of TB were estimated to occur each year, accounting for 14.3% of incidental TB globally [1]. Extrapulmonary tuberculosis contributed to 9.2% to 11.2% of active tuberculosis in China [2], and the percentage increased to 23.5% in children [3].

Tuberculous serositis, including tuberculous pleuritis, peritonitis and pericarditis, is a common form of extrapulmonary tuberculosis and common cause of serous effusion, especially in high TB endemic settings [4]–[6]. Culture of serous effusion or tissue biopsy specimens has been considered as the gold standard for the diagnosis of tuberculous serositis. However, several disadvantages, including long time lag, poor sensitivity and invasive operation, render this diagnostic method unsuitable for routine practice. The sensitivity of pleural effusion culture was only 63% from a study in Taiwan, although the diagnostic yield was higher than previous reports. For pleural biopsy, similar test sensitivity (74%) was reported [7].

Interferon-gamma release assay (IGRA), which detects interferon γ responding to the *Mycobacterium tuberculosis* (MTB) specific antigens encoded in the RD1 region, has been developed as a sensitive, specific and rapid immunodiagnostic test for TB infection. However, the sensitivity of IGRA on blood samples varies according to the site of infection [8], [9]. Additionally, it cannot accurately differentiate active tuberculosis (ATB) from latent tuberculosis infection (LTBI), and as a result, has a reduced specificity for diagnosis of ATB in high burden settings where LTBI is prevalent.

Several studies have evaluated the diagnostic value of IGRA in patients with tuberculous pleuritis. The sensitivities of IGRAs using pleural fluid as test samples were ranging from 86.4–100% for T-SPOT.TB [10]–[13], and 44.4–96.4% for QFT-G[14]–[16]. Unfortunately, these studies were limited by small sample size, which reduced the generalizability of their results. In addition, few studies have investigated the diagnostic value of IGRA for tuberculous peritonitis and pericarditis, using serous cavity fluid as test samples, or have compared their performance with tests based on blood samples.

The aims of the present study are to conduct a prospective cohort study in a high TB burden area to evaluate the diagnostic accuracy of T-SPOT.TB on serous effusion mononuclear cells (SEMC) for HIV-negative adult patients with tuberculous serositis, and to discuss the optimal cut-off value of T-SPOT.TB on SEMC for diagnosis of tuberculous serositis.

## Methods

### Ethics statement

This study was approved by the Ethics Committee of Peking Union Medical College Hospital. Written informed consent was obtained from all patients enrolled in this study.

### Patients and study procedures

A prospective study was conducted in Peking Union Medical College Hospital in China from June 2008 to September 2011. All adult patients (>15 years) admitted into this hospital with suspected tuberculous serositis were considered and were included into the study if serous cavity fluid (pleural effusion, ascites, pericardial effusion) were obtained. Patients were evaluated with routine diagnostic work-up according to their presentations. Clinical information was extracted from patients' medical records by researchers blinded to the T-SPOT.TB results, who also tracked patients' treatment process and discharge diagnosis. For patients whose diagnosis was not established during hospitalization, a telephone interview was conducted 3–6 months later to obtain the diagnosis. At the end of follow-up, each case was classified into one of predefined clinical categories, including culture-confirmed tuberculosis, highly probable tuberculosis, clinically indeterminate and active tuberculosis excluded, based on the clinical, radiological, microbiological, pathologic information and response to anti-TB therapy (Table 1).

**Table 1:** Categorization of the Study Population.

Diagnostic Category	Criteria
1.Tuberculous serositis	
1) Culture-confirmed TB	Acid-fast stain or culture positive for MTB, OR typical histologic changes
	(caseousnecrosis, epithelioid granuloma, etc.)AND
	Suggestive clinical and radiologic findings
2) Highly probable TB	Clinical manifestations, laboratory results and radiologic features highly
	suggestive of tuberculous serositis AND
	Appropriate response to anti-TB therapy
2.Clinically indeterminate	A final diagnosis of tuberculous serositis was neither highly probable
	nor reliably excluded
3.Tuberculous serositis excluded	All microbiological samples smear and culture negative AND
	A definite alternative diagnosis identified AND
	Effective treatment of primary disease

Fifty milliliter of serous fluid and 4 ml of peripheral blood were collected from each patient. Specific T cell responses to RD1 encoded antigens were detected by T-SPOT.TB (Oxford Immunotec, Abingdon, UK) that was performed within 6 hours from sample collection by laboratory personnel blinded to patients' clinical data. T-SPOT.TB utilized AIM-V (GIBCO™ AIM V Medium liquid, Invitrogen, US.) as negative control, PHA as positive control, and ESAT-6 and CFP-10 as specific antigens, respectively. Serous effusion mononuclear cells (SEMC) were separated by Ficoil-Hypaque gradient centrifugation. SEMC and PBMC obtained from each subject were plated (2.5×10^5^ per well) on a plate precoated with antibody against interferon γ. Plates were incubated 16-18 h at 37°C in 5% carbon dioxide. After incubation, wells were developed with a conjugate against the antibody used and an enzyme substrate. Spot-forming cells (SFCs) were counted with an automated ELISPOT reader (AID-ispot, Strassberg, Germany), each SFC represented an antigen-specific T cell secreting interferon γ. The response was considered positive when the antigen well contained 6 or more spots and had twice the number of spots than the negative control well. The background number of spots in negative control well for PBMC and SEMC should be less than 10 spots and 20 spots, respectively.

### Statistical and Data Analysis

Sensitivity, specificity, positive predictive value (PPV), negative predictive value (NPV), likelihood ratio positive (LR+), and likelihood ratio negative (LR-) were calculated to evaluate diagnostic performance for the T-SPOT.TB on SEMC and PBMC. The area under the receiver operating characteristic curve (AUC) of the T-SPOT.TB on SEMC diagnostic cutpoint for tuberculous serositis were calculated. The difference in means was assessed using Students' t-tests. The Pearson's Chi-square test was used to compare the positive proportions. Ninety five percent confidence intervals (95%CI) were estimated according to the binomial distribution. Significance was inferred for P<0.05. Statistical analysis was performed by SPSS16.0.

To compare the diagnostic sensitivities of T-SPOT.TB on PBMC and that on SEMC, the sample size was determined by the following formula. We assumed that the sensitivity of T-SPOT.TB on PBMC was 70%, the sensitivity of T-SPOT.TB on SEMC was 95%, type I error was 0.05 and power was 0.90. According to these assumptions, the minimum sample size was 205[17].

## Results

206 HIV-negative hospitalized patients with suspected tuberculous serositis were prospectively enrolled into this study. 13 participants were excluded from analysis due to indeterminate T-SPOT.TB results (4 samples of blood and 11 samples of serous cavity effusion), and 6 patients were excluded due to lost to follow up and indeterminate diagnosis. Data of 187 participants were analyzed, whose demographic characteristics are shown in Table 2. Tuberculous serositis was diagnosed in 74 patients (39.6%), among whom 7 were culture or histology confirmed cases. Twenty (10.7%) were classified as clinically indeterminate. Tuberculous serositis was excluded in 93 patients (49.7%), among whom the diagnosis included hematological or solid malignancy, autoimmune disease, infectious diseases, and other diseases. Age, sex, use of corticosteroid or immunosuppressive agents, previous history of tuberculosis, sites of serositis, and pre-existing conditions were not significantly different between three groups.

**Table 2:** Demographic and characteristics of the study population.

Characteristic	Tuberculous	Clinically	Tuberculous	Total
	serositis	indeterminate	serositis excluded	
Total	74	20	93	187
Age(years), (median, IQR)	56[35–67]	65[35–78]	54[38–67]	54[34–69]
Gender				
Male(%)	35(47.3%)	10(51.9%)	47(50.5%)	92(49.2%)
Female(%)	39(52.7%)	10(48.1%)	46(49.5%)	95(50.8%)
Under immunosuppressive condition(%)	11(14.9%)	7(35.0%)	18(19.4%)	36(19.3%)
Evidences of previous TB (%)	10(13.5%)	7(35.0%)	8(8.6%)	25(13.5%)
Contact history of pulmonary TB(%)	11(14.9%)	3(15.0%)	7(7.5%)	21(11.2%)
Source of serous effusion				
Pleural effusion(%)	45(60.8)	14(70.0)	49(52.7)	108(57.8)
Peritoneal effusion(%)	18(24.3)	6(30.0)	32(34.4)	56(29.9)
Pericardial effusion(%)	11(14.9)	0	12(12.9)	23(12.3)
Pre-existing conditions	7(21.9%)	4(57.1%)	65(69.6%)	20(26.7%)

PBMC: peripheral blood mononuclear cell; SEMC: serous effusion mononuclear cells.

PPV: positive predictive value; NPV: negative predictive value; LR: likelihood ratio.

Among 74 patients with tuberculous serositis (confirmed and probable), 68 were positive by T-SPOT.TB on SEMC with a sensitivity of 91.9%(95% confidence interval [CI]: 78.8%–92.5%), significantly higher than the sensitivity of T-SPOT.TB on PBMC (P = 0.002). For 7 confirmed cases, both T-SPOT.TB on SEMC and PBMC have a sensitivity of 100%. Among 93 patients excluded tuberculous serositis, 81 and 68 were non-reactive by T-SPOT.TB on SEMC and PBMC, respectively. The difference of specificities was statistically significant (P = 0.017). The concordance between T-SPOT.TB results on SEMC and PBMC was evaluated in 167 patients with definitive diagnosis, and the agreement was 79%(Kappa = 0.58, P<0.001).

The sensitivity of T-SPOT.TB in pleural, peritoneal and pericardial effusion was 88.9%, 94.4% and 100%, respectively. The difference was not significant (P = 0.281). And there was no significant difference of specificity in serous fluids from different sources (85.7%, 87.5% and 91.7%, respectively. P = 0.777).

We also calculated the predictive values (PV) and likelihood ratios (LR) for each test in 167 patients with definitive diagnosis. 68 out of 80 patients with positive results of T-SPOT.TB on SEMC were diagnosed as tuberculous serositis, which was compared to 54 out of 79 patients with reactive results of T-SPOT.TB on PBMC who were diagnosed as tuberculous serositis. The positive predictive value of T-SPOT.TB on SEMC was significantly higher than that on PBMC (85.0% vs. 68.4%, P = 0.013). Negative predictive value of T-SPOT.TB on SEMC was also significantly higher than that on PBMC (93.1% vs. 77.3%, P = 0.003)(Table 3). The PLR and NLR of T-SPOT.TB on SEMC were 7.12 and 0.09, respectively, both of which were significantly higher than those on PBMC (Table 3).Combined PPV of serial tests was 87.1%(95%CI: 76.6%–93.3%), and combined LR+ of serial tests increased to 8.48(95%CI: 4.31–16.6).When T-SPOT.TB on PBMC was serially combined with SEMC, the specificity rose to 91.4%(95% CI: 83.9%–95.6%).However, combined sensitivity of parallel test was not improved(91.9%, 95%CI: 83.4%–96.2%).

**Table 3:** Single and combined diagnostic parameters of T-SPOT.TB on SEMC and PBMC.

	Sensitivity (95%CI)	Specificity (95%CI)	PPV (95%CI)	NPV (95%CI)	LR+ (95%CI)	LR- (95%CI)
T-SPOT.TB on PBMC	73.0% (61.9–81.8)	73.1% (63.3–81.1)	68.4% (57.5–77.6)	77.3% (67.5–84.8)	2.72 (1.89–3.90)	0.37 (0.25–0.55)
T-SPOT.TB on SEMC	91.9% (83.4–96.2)	87.1% (78.8–92.5)	85.0% (75.6–91.2)	93.1% (85.8–96.8)	7.12 (4.18–12.13)	0.09 (0.04–0.20)
T-SPOT.TB on PBMC & SEMC (parallel)	91.9% (83.4–96.2)	68.8% (58.8–77.3)	70.1% (60.4–78.3)	91.4% (82.5–96.0)	2.95 (2.16–4.02)	0.12 (0.05–0.26)
T-SPOT.TB on PBMC & SEMC (serial)	73.0% (61.9–81.8)	91.4% (83.9–91.6)	87.1% (76.6–93.3)	81.0% (72.4–87.3)	8.48 (4.31–16.69)	0.30 (0.20–0.43)

Parallel and serial testing algorithms [18] appeared more accurate than single T-SPOT.TB on PBMC, but T-SPOT.TB on SEMC. The serial testing increased the specificity of T-SPOT.TB on SEMC from 87.1% up to 91.4%, and increased PLR from 7.12 up to 8.48(Table 3).

In the sensitivity analysis, when indeterminate results were treated as negative, the sensitivity, specificity, PPV and NPV for T-SPOT.TB of serous effusion mononuclear cells in diagnosis of TB serositis were 90.8%, 88.2%, 85.2% and 92.8% respectively. When indeterminate results were treated as positive, the sensitivity, specificity, PPV and NPV were 92.1%, 79.4%, 76.9%, and 93.1% respectively.

**Figure 1:**
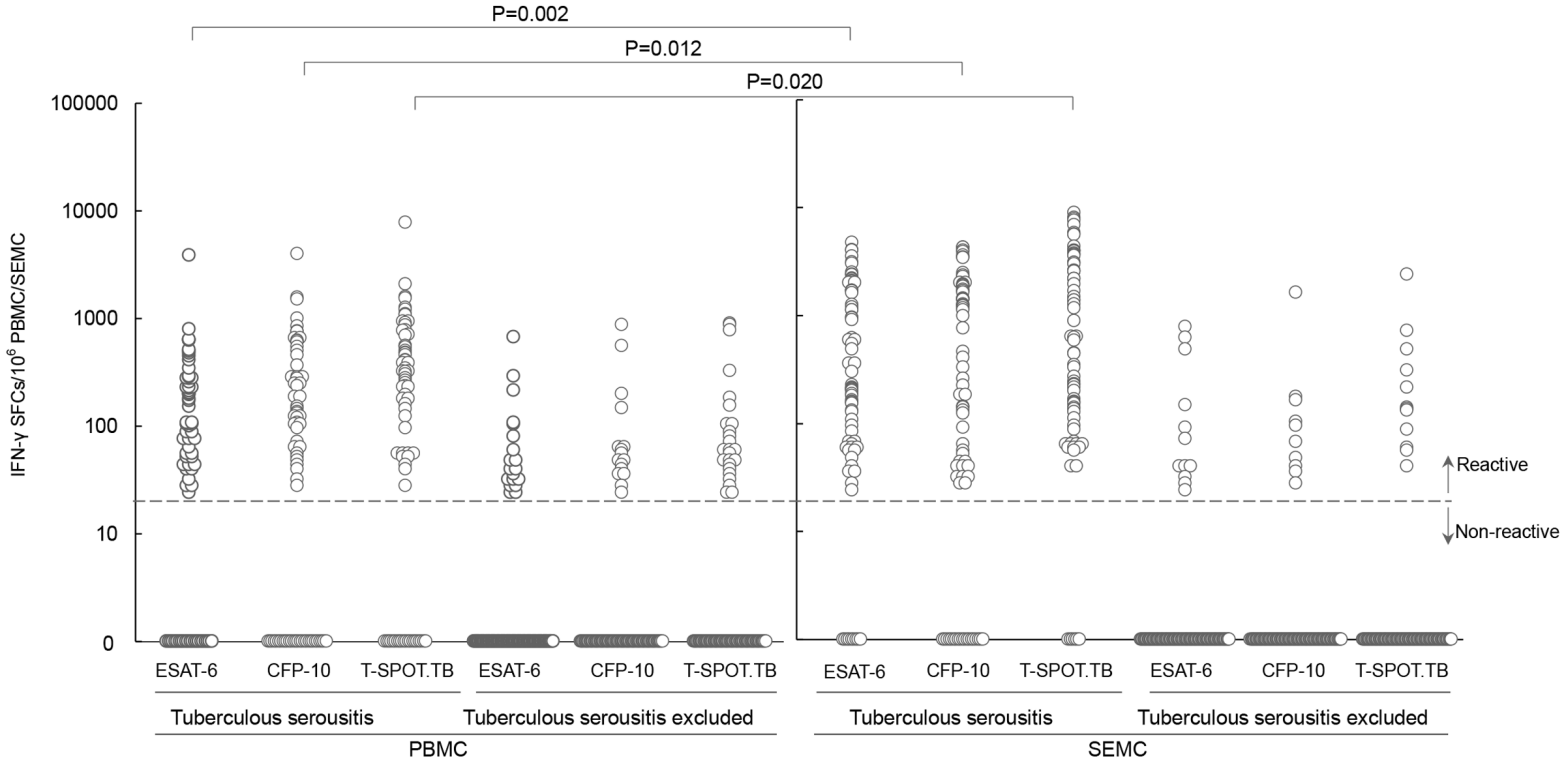
Frequencies of MTB-specific IFN-γ secreting T cells in serous effusion and peripheral blood. The frequencies of ESAT-6 and CFP-10 specific IFN-γ secreting T cells in SEMC were significantly higher than those in PBMC (P = 0.002 for ESAT-6, P = 0.012 for CFP-10). The counts of IFN-γ secreting T cells specific for CFP-10 appeared higher than ESAT-6, but the difference were not statistically significant (P = 0.573 for serous effusion, P = 0.092 for peripheral blood). PBMC: peripheral blood mononuclear cell; SEMC: serous effusion mononuclear cells

For 74 patients with tuberculous serositis (confirmed and probable), the median count of antigen-specific IFN-γ secreting T cells in serous cavity effusion samples were 636 SFCs/million SEMC (interquartile range [IQR]: 143–3443), which were significantly higher than those in peripheral blood samples (P = 0.002). The frequencies of spot forming cells for T-SPOT.TB were 4.6-fold (IQR: 1.3–14.3) higher in SEMC than in PBMC (4.4-fold for ESAT-6, 3.9-fold for CFP-10). The frequencies of ESAT-6 and CFP-10 specific IFN-γ secreting T cells in SEMC were significantly higher than those in PBMC (P = 0.002 for ESAT-6, P = 0.012 for CFP-10, Fig.1). The counts of IFN-γ secreting T cells specific for CFP-10 appeared higher than ESAT-6, but the difference were not statistically significant (P = 0.573 for serous effusion, P = 0.092 for peripheral blood). (Fig.1)

**Figure 2:**
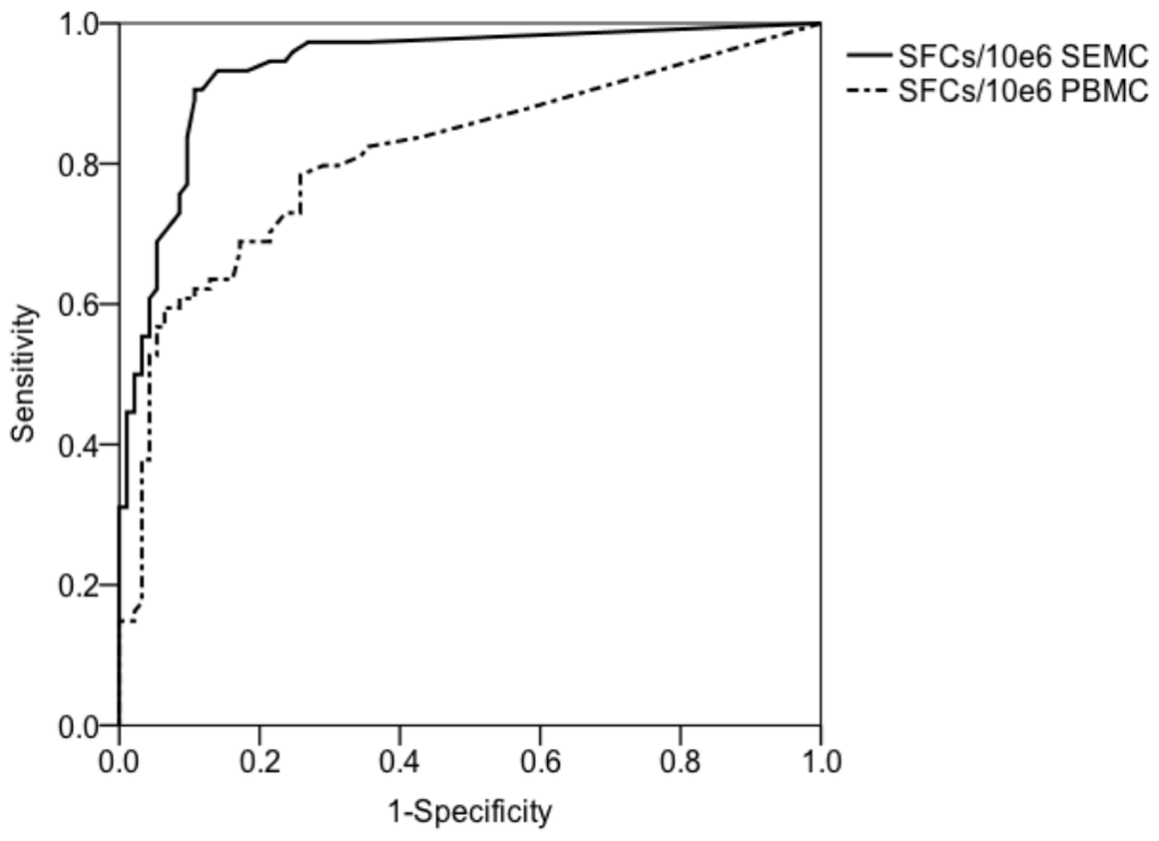
ROC curves for T-SPOT.TB on SEMC and PBMC in patients with suspected tuberculous serositis. The AUC of ROC curve was 0.938 (95%CI: 0.900–0.975,P<0.001) for T-SPOT.TB on SEMC, which was higher than that of T-SPOT.TB on PBMC (0.811,95%CI: 0.742–0.880,P<0.001). PBMC: peripheral blood mononuclear cell; SEMC: serous effusion mononuclear cells; ROC: receiver operating characteristic; AUC: area under the receiver operating characteristic curve.

ROC curves were used to estimate the diagnostic values of T-SPOT.TB on SEMC and PBMC in 167 patients with confirmed TB or TB excluded. The frequencies of spot forming cells for ROC curves were sum of ESAT-6- and CFP-10- specific IFN-γ secreting T cells. The AUC of ROC curve was 0.938 (95%CI: 0.900–0.975,P<0.001) for T-SPOT.TB on SEMC, which was higher than that of T-SPOT.TB on PBMC (0.811,95%CI: 0.742–0.880,P<0.001)(Fig.2). Based on ROC curve analysis, the cutoff value for the diagnosis of tuberculous serositis was 56 SFCs/10^6^ for T-SPOT.TB on SEMC, with a sensitivity of 90.5%, a specificity of 89.2%. The PPV was 87.0%, with NPV of 92.2%, PLR of 8.38, and NLR of 0.11.

## Discussion

This is a prospective study to evaluate the utility of T-SPOT.TB on SEMC for the diagnosis of tuberculous serositis in TB endemic settings and explore the cut-off value to diagnose tuberculous serositis.

Many studies demonstrated that MTB-specific IFN-γ response in ATB patients was significantly higher than patients with LTBI, since the intensity of response was possibly associated with the bacterial burden. However, there were some ATB patients having weaker response than the level seen in LTBI, which might be due to immune anergy. Compartmentalization of antigen-specific T cells in pleural effusion had been demonstrated previously [19], and the mechanisms of T-lymphocyte accumulation in the pleural space were investigated in a murine model. Level of many cytokines such as IFN-γ was markedly elevated in pleural effusion, compared to peripheral blood in patients with tuberculous pleuritis. The accumulation of MTB-specific T cell in pleural effusion results from selectin-mediated migration and a local proliferation [20]. The concentration of MTB-specific IFN-γ secreting T cells at the site of infection made it possible to diagnose tuberculous serositis efficiently by using T-SPOT.TB on SEMC instead of PBMC.

A study from UK first demonstrated that enumeration of MTB-specific mononuclear cells from pleural effusion could improve the diagnosis of tuberculous pleuritis. Besides, MTB-specific T cells had higher frequencies and broader repertoires at the site of disease than the peripheral blood [10]. In a multicenter study for the diagnosis of tuberculous pleuritis, compared to T-SPOT.TB on PBMCs, that on PEMCs improved the sensitivity from 76% to 95% and the specificity from 67% to 95%[11]. A study in Taiwan showed that the sensitivity and specificity of T-SPOT.TB were 94.7% and85.7%on pleural fluid, and 77.8% and90.5%on blood. However, the differences between T-SPOT.TB on pleural fluid and blood were not significant, perhaps due to the small sample size of the study [12]. A study in South Africa revealed that the sensitivity of pleural T-SPOT.TB was 86%, while the specificity was only 60%, both of which were similar to the commercial T-SPOT.TB test [13].

In our study, both the sensitivity and specificity of T-SPOT.TB on SEMC were significantly higher than the test on PBMC, for diagnosis of tuberculous serositis. The sensitivity of T-SPOT.TB on PBMC in diagnosing tuberculous serositis was 73%, lower than that in extrapulmonary tuberculosis from previous studies. One possible reason was the migration of MTB-specific effector T cells from peripheral blood to serous cavity, which decreased the density of IFN-γ secreting T cells in blood [20]. In our study, there were 22 patients with tuberculous serositis and tuberculosis of other sites (intestinal tuberculosis, renal tuberculosis),20 of whom were reactive in T-SPOT.TB on PBMC(90.9%), which appeared comparable with previous studies[21]. In high TB burden countries like China, prevalent LTBI inevitably lead to a reduced specificity of T-SPOT.TB on PBMC for diagnosis of ATB. However, with T-SPOT.TB using SEMC instead of PBMC, the specificity could be improved to 87.1%, indicating a more accurate diagnostic option.

In our study, T-SPOT.TB on SEMC were found false negative in 6 patients, including one hemodialysis patients with chronic renal failure, three patients having received anti-TB therapy for more than 4 weeks. The anti-TB therapy probably affected the sensitivity of T-SPOT.TB on SEMC as well as that on PBMC [22]. Twelve patients got false positive results in serous effusion, including seven patients with malignant tumors, four patents with autoimmune disease and one patient with inflammatory bowel disease, which indicated that aberrant immune activation might influence the diagnostic accuracy.

Both the predictive value and likelihood ratio of T-SPOT.TB on SEMC were higher than those on PBMC in diagnosing tuberculous serositis. The NPV was 93.1% and NLR was 0.09, indicating that nonreactive results would be helpful in excluding tuberculous serositis. We also evaluated diagnostic performance of T-SPOT.TB on SEMC and PBMC combined in parallel in serial orders. The serial combination brought the specificity and PLR of T-SPOT.TB on SEMC up to 91.4% and 8.48, respectively. However, the parallel combination test did not improve diagnostic efficiency significantly. The combination of T-SPOT.TB on SEMC and PBMC was recommended for two reasons in our study. Firstly, the ratio of SFCs in SEMC to PBMC was helpful in diagnosing tuberculous serositis, as the frequencies of IFN-γ secreting T cells in serous effusion were 4.6 times of those in peripheral blood. Secondly, the results of T-SPOT.TB on SEMC were more likely than PBMC to be indeterminate (Nil control>20 spots). There were 13 indeterminate results of T-SPOT.TB, 11 of which came from serous cavity effusion.

For diagnosis of active and latent TB infection, T-SPOT.TB approved by FDA recommended a 24-SFCs/10^6^PBMC cutoff for peripheral blood sample, while the cut-off value may not be suitable for detecting mononuclear cells in serous cavity effusion. To our knowledge, this is the first prospective study to explore the optimal cut-off value for T-SPOT.TB on SEMC for diagnosing tuberculous serositis. About 90% of subjects latently infected with M. tuberculosis would never develop ATB, indicating that the host immune response was capable of controlling MTB infection effectively. However, the host immunity failed to eradicate tubercle bacilli successfully,and the risk of reactivation increased with immunosuppression and bacterial replication. This was accompanied by increased MTB-specific antigen responses. Base on this assumption, a cut-off value of frequencies of MTB-specific IFN-γ secreting cells could be established to diagnose active tuberculosis [23]. Studies have shown that the magnitudes of IFN-γ response in ATB patients were significantly higher than that in LTBI subjects, but there was considerable overlap between the two groups, which limited the discriminatory ability of IGRAs, especially in high endemic settings such as China [24]–[26]. As to T-SPOT.TB on PBMC, our study showed the same result as previous studies that it was difficult to give an ideal cut-off value for diagnosis of tuberculous serositis. However, both sensitivity and specificity of T-SPOT.TB on SEMC could be increased by about 30% when using 56 SFCs/10^6^SEMC as the cut-off value for active disease. Our study demonstrated that T-SPOT.TB on SEMC significantly improved diagnostic efficiency, especially specificity, in high tuberculosis burden areas.

Previously, few studies have reported on the diagnostic accuracy of T-SPOT.TB with pericardial effusion mononuclear cells for tuberculous pericarditis, although limited case reports have suggested the diagnostic potential of ELISPOT assay in tuberculous pericarditis [27]–[29]. Our study included 23 patients with pericardial effusion. Among 11 patients with tuberculous pericarditis, all of them were positive for T-SPOT.TB on SEMC (100%),compared to 7 positive for T-SPOT.TB on PBMC(63.6%).Among 12 patients with tuberculous pericarditis excluded, 11 were negative in T-SPOT.TB on SEMC(91.7%), compared to 9 negative on PBMC. These data indicated that T-SPOT.TB with pericardial effusion mononuclear cells was a useful method to diagnose tuberculous pericarditis. Though the sensitivity and specificity of T-SPOT.TB in different source of serous effusions were not significantly different, the indeterminate results were more likely to happen in peritoneal effusion.

Our study has two major limitations. First, many patients with tuberculous serositis were diagnosed according to clinical criterion rather than culture-confirmed. We included highly probable cases to better reflect real clinical situations. But it would likely underestimate or overestimated the sensitivity and specificity of T-SPOT.TB assays in this study. Second, we do not evaluate the utility of TST for diagnosing tuberculous serositis, because it would lead to considerable false positive cases in areas where BCG vaccination is widely performed. And we do not exclude participants with a preceding TST prior to a T-SPOT.TB, which may induce boosting effect of IGRA response.

In conclusion, we found that T-SPOT.TB on SEMC was an accurate diagnostic method for tuberculous serositis. Based on our study, 56 SFCs/10^6^ SEMC may be optimal cut-off value for T-SPOT.TB on SEMC to diagnose tuberculous serositis.
